# Rheologic Behavior of Bovine Calf Serum

**DOI:** 10.3390/ma14102538

**Published:** 2021-05-13

**Authors:** Tanja Wonerow, Maximilian Uhler, Jens Nuppnau, J. Philippe Kretzer, Frank Mantwill

**Affiliations:** 1 Department of Mechanical Engineering, Machine Elements and Computer-Aided Product Development, Helmut-Schmidt-University, 22043 Hamburg, Germany; jens.nuppnau@hsu-hh.de (J.N.); frank.mantwill@hsu-hh.de (F.M.); 2 Laboratory of Biomechanics and Implant Research, Clinic for Orthopedics and Trauma Surgery, Heidelberg University Hospital, Heidelberg University, 69118 Heidelberg, Germany; philippe.kretzer@med.uni-heidelberg.de

**Keywords:** biomedical rheology, viscosity, bovine calf serum, shear thinning, wear, joint replacement, arthroplasty, numerical simulation

## Abstract

Recent studies have illuminated the rheological behavior of synovial fluid and the role of protein and hyaluronan (HA). However, with respect to artificial joint replacement in standardized wear simulations, bovine serum is used as fluid test medium. Little is known about the rheological characteristics of bovine serum, which are needed for precise tribological investigations. The steady shear viscosity η of bovine calf serum is determined for protein concentrations used in standardized wear simulations depending on shear rate $\dot{\gamma }$ and temperature *T*. Additionally, the density of the serum is determined for both protein concentrations. The results show shear thinning behavior of bovine calf serum with a nearly Newtonian behavior in the range of high shear rates. Within the range of high shear rates, mean viscosities of *η* = 0.82–0.88 mPa·s were found for protein concentrations of 20 g/L and mean viscosities of *η* = 0.88–0.94 mPa·s for 30 g/L, decreasing with temperature. Densities of 1.004–1.005 g/cm^3^ and 1.007–1.008 g/cm^3^ were found for 20 and 30 g/L protein concentrations, respectively.

## 1. Introduction

Joint replacement in the main joints of the human body, such as the hip, knee or shoulder joints, has become increasingly important. In 2019, 315,088 interventions in the field of joint replacement were registered in Germany. In total, 89.6% of these procedures were primary implantations [1]. In addition to degenerative joint diseases such as osteoarthritis and arthrosis [2], high stresses [3] and a frequent overstraining of the joint [4], which leads to continuous changes and wear in the cartilage [2], are the main reasons for joint replacement. The aim of endoprosthetic replacement is to restore the functionality of the joint and to ensure painless mobility of the patient [5]. However, even with joint replacement, aseptic and septic loosening can lead to implant failure and revisions. These kinds of failures are generally multifactorial, but the main reasons may be related to mechanical or biological causes [6]. The removal of wear particles from the implant material clearly plays a crucial role [7]. The consequences for the joint differ depending on the amount, size, shape and chemical composition of the wear products of artificial joints [8,9,10,11,12,13].

For these reasons, tribological examination of the implants used in total joint replacements plays an essential role [14]. The focus of these investigations is to characterize friction and wear behavior under in vitro conditions and to examine the biological response of the wear products [15]. Controlled wear testing of joint replacements is well established and offers the possibility to give a prediction of long-term clinical performance [16,17,18].

Nevertheless, the in vitro test cannot fully reflect the in vivo conditions. In some investigations, results have already been achieved that cannot correspond to the in vivo situation [19,20,21]. One of the main reasons for these deviations could be the change in mechanical properties caused by the used test fluid [22,23].

Computer simulations may help to understand the process of tribological behavior of artificial joints and to optimize the surface geometry with respect to friction and wear reduction. However, to achieve valid results, the rheological properties of the used fluid must be known. For human synovial fluid, a non-Newtonian shear thinning behavior is usually observed [24,25,26]. Common rheological models to simulate this kind of non-linearity are the Cross and Carreau models, which are also able to model zero-shear viscosity and infinite shear viscosity [27,28].

In wear simulations standardized, for example, according to ISO 14242 and ISO 14243, bovine serum is used as fluid test medium [29,30]. The protein concentration and other additives are defined in these standards to imitate human synovial fluid as well as possible. For standardized wear tests of knee endoprostheses, a protein concentration of 20 g/L is used [30]. A concentration of 30 g/L is prescribed for examinations concerning the hip joint [29]. While several studies exist regarding the rheological characterization of human synovial fluid [24,25,26], few could be found for bovine serum. The objective of this work is the rheological characterization of bovine serum used in standardized wear simulations of artificial joints. In addition, the influence of shear rate, protein concentration and temperature on rheological behavior will be considered.

## 2. Materials and Methods

### 2.1. Bovine Calf Serum

In order to obtain properties corresponding to the joint, the bovine calf serum (BCS) (Biochrom GmbH, Berlin, Germany) was first diluted to the appropriate protein content by the addition of deionized water. A protein content of 20 g/L is specified for knee and shoulder joint replacements [30] and 30 g/L for the hip joint replacements [29]. In total, 1.85 g/L sodium azide (NaN_3_) and 5.85 g/L ethylenediaminetetraacetate (EDTA) were added as anti-microbiological reagents [29,30]. The used BCS was tested for viruses (BVDV, BHV-1, IBRV, PIV-3) and endotoxins. After preparation, all samples were pre-cooled to 4 °C and were deep-frozen afterwards at −20 °C.

### 2.2. Density Measurements

Density measurements were performed using a DMA 4500 density meter (Anton Paar GmbH, Graz, Austria). Density measurements were carried out for protein concentrations of 20 and 30 g/L, which were used in standardized joint wear simulations.

### 2.3. Rheologic Behavior

The rheologic behavior of BCS was determined using a Physica MCR 702 rheometer (Anton Paar GmbH, Graz, Austria). The rheometer was air bearing driven with a torque range of 1 nNm to 230 × 10^6^ nNm. The rheometer was equipped with a double gap measuring system with a cup outer diameter of 27.596 mm and a Peltier temperature control. The material of the measuring tools is stainless steel. A covering hood was employed to avoid evaporation.

Before the measuring cup was filled, the fluid to be tested was thoroughly mixed by swirling it gently. The test was started after the temperature was maintained with a deviation of ±0.04 °C for 60 s. Dynamic viscosity η was measured as a function of the shear rate $\dot{\gamma },$ covering a range from 1 to 1000 s^−1^, ramping up in 22 logarithmic steps starting with low shear rates. The measurement duration varied from 240 to 0.5 s during this process. Previously, the fluid was rotated for 30 s at a shear rate of 50 s^−1^ for thorough mixing and then was stopped again for 10 s. This was carried out to diminish the sedimentation rate of the proteins and other components and counteract their deposition as the temperature control takes a while. Between each test, the measuring tools were thoroughly cleaned with water and isopropyl alcohol.

Measurements were performed for protein concentrations of 20 and 30 g/L at three biologically relevant temperatures of 35, 37 and 39 °C each. Each setting point was measured *n* = 10 times using fresh samples. Furthermore, the quality of the measurements was verified by preliminary tests with deionized water at 30 °C. A temperature of 30 °C was chosen since the viscosity of water at this temperature ($\eta_{water,30\;{}^{\circ}C}=0.8\;\mathrm{mPa}\cdot s$) is in the range of obtained viscosities for BCS at high shear rates.

In addition, the Reynolds number was calculated for all measurements to ensure laminar flow for the entire shear rate range. In the annular gap of a cylinder measuring system, the Reynolds number was calculated, in general, as

$$Re=\frac{v_{m}L\rho }{\eta }$$

With the density ρ and the viscosity η of the fluid, the annular gap L and the velocity $v_{m}$ refer to the gap center. The annular gap results from the difference of the outer and inner radius.

$$L=R_{e}-R_{i}$$

The velocity referring to the gap center was determined as follows

$$v_{m}=\omega \frac{R_{e}+R_{i}}{2}$$

with ω being the angular velocity [31].

### 2.4. Cross Model

The shear rate dependency of the dynamic viscosity was fitted by the Cross model [32], given as

$$\eta (\gamma )=\eta_{\infty }+\frac{\eta_{0}-\eta_{\infty }}{1+(C\cdot {\gamma )}^{m}}$$

With the zero-shear viscosity $\eta_{0}$, the infinite shear viscosity $\eta_{\infty }$, the Cross time constant *C* and the Cross rate constant *m*. The four Cross constants were fitted following the rheological models suggested in literature for bovine serum [33] and human synovial fluid [27,28].

### 2.5. Statictical Analysis

For statistical analysis, the software SPSS 25 (IBM Inc., Armonk, NY, USA) was used. A descriptive analysis (arithmetic mean, standard deviation, 95% confidence interval) was performed. With the Shapiro–Wilk test, the normal distribution of the data was confirmed. Therefore, a repeated measures ANOVA of independent variables was applied. To compare the groups, an ANOVA with a Bonferroni correction was used. A *p*-value of <0.05 was considered significant. In addition, the intra-class-correlation coefficient was calculated between the measured values for concentrations of 20 and 30 g/L at 37 °C and the theoretical values from the Cross model of these measurements. Here, a two-way mixed intra-class-correlation with absolute agreement was used.

## 3. Results

### 3.1. Density Measurement

In Table 1, the results of the density measurements are given. Protein concentrations of 20 and 30 g/L, which were used in standardized wear simulations, and the undiluted raw serum with a protein concentration of 70 g/L were investigated. The density varied between 1.0037 and 1.0208 g/cm^3^ depending on the concentration and temperature. For the higher protein concentration, a higher density was found, while with increasing temperature, a slightly decrease in the density could be seen.

**Figure 1:**
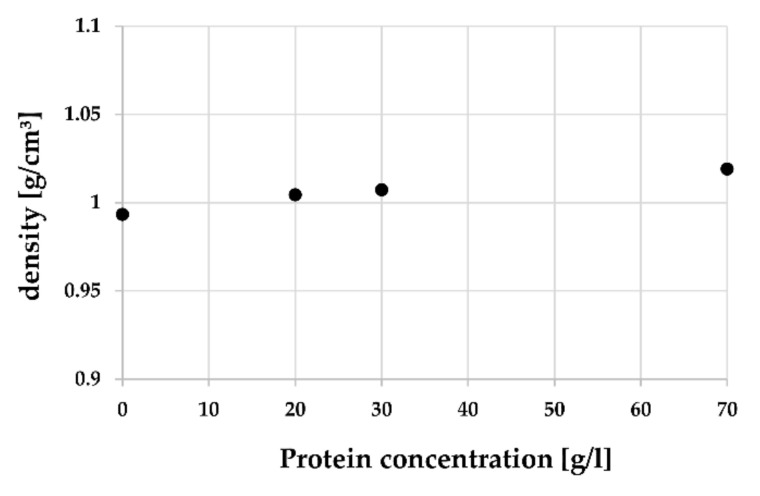
Density of BCS in relation to protein concentration at 37 °C, including the density of water (0 g/L).

Figure 1 shows the measured density of BCS in relation to the protein concentration at 37 °C. For comparative purposes, these values are presented in relation to the density of deionized water at 37 °C, which would equal a protein concentration of zero.

### 3.2. Rheologic Behaviour

**Figure 2:**
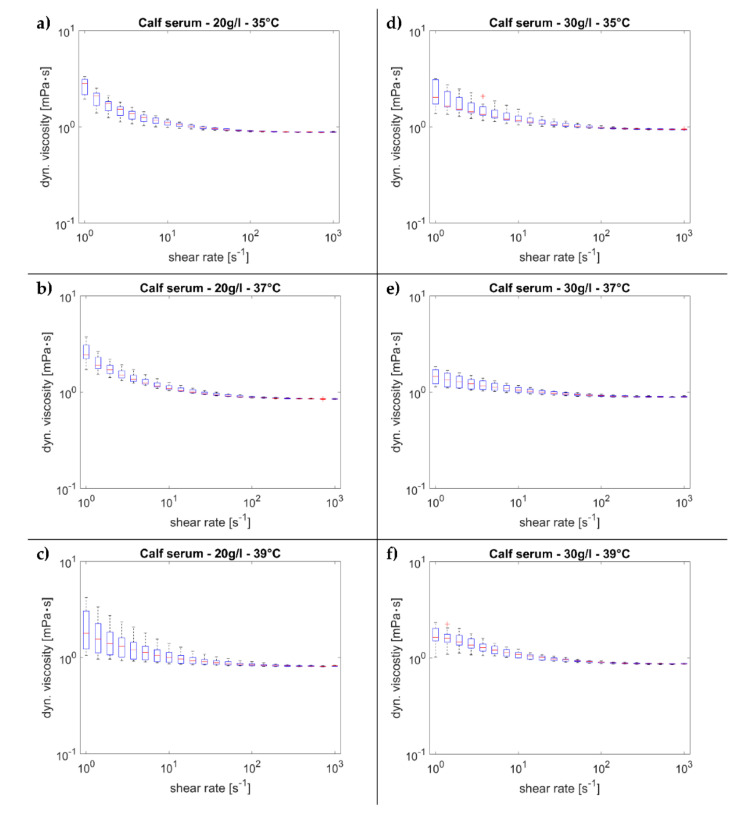
Dynamic viscosity over shear rate. (**a**) Conc.—20 g/L, 35 °C; (**b**) conc.—20 g/L, 37 °C; (**c**) conc.—20 g/L, 39 °C; (**d**) conc.—30 g/L, 35 °C; (**e**) conc.—30 g/L, 37 °C; (**f**) conc.—30 g/L, 39 °C; ⁺ = outliners.

In Figure 2, the results of the dynamic viscosity measurements are given as boxplots. On the left side, the results for a protein concentration of 20 g/L are shown and on the right side the results for protein concentration of 30 g/L are shown. Overall, a shear thinning behavior can be seen merging into nearly Newtonian behavior at high shear rates ($\dot{\gamma }$ ≥ 100 s^−1^). For low shear rates, a clearly higher deviation of the measured values can be seen than that for high shear rates. The highest deviation occurred for a protein concentration of 30 g/L at a temperature of 39 °C.

**Figure 3:**
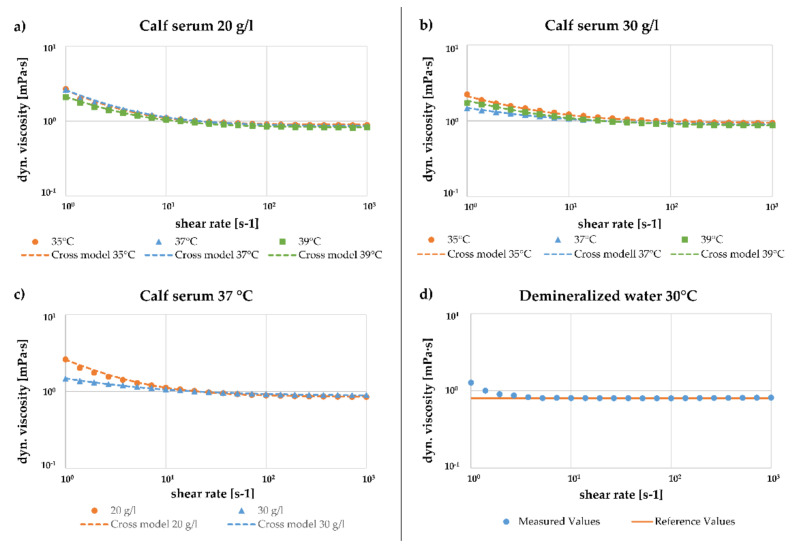
Comparison of the mean dynamic viscosity over shear rate with a fit to the Cross model. (**a**) At different temperatures for 20 g/L; (**b**) at different temperatures for 30 g/L; (**c**) different protein concentrations at 37 °C; (**d**) comparison measurement of water at 37 °C and reference values.

Figure 3a,b show the comparison of the mean dynamic viscosity curves depending on the shear rate at different temperatures for protein concentrations of 20 and 30 g/L. While for shear rates $\dot{\gamma }$ ≥ 100 s^−1^ a clear tendency of decreasing viscosity with increasing temperature can be seen, this trend cannot be noticed at lower shear rates. In the range of low shear rates, the highest viscosity occurs at a concentration of 20 g/L at 37 °C, whereas for a protein concentration of 30 g/L at a temperature of 37 °C, the averaged viscosities were found being the lowest.

A similar effect can be seen when comparing the mean dynamic viscosity curves of different protein concentrations at a constant temperature of 37 °C (Figure 3c). In the range of low shear rates, the mean dynamic viscosity is higher for a protein concentration of 20 g/L, while in the range of high shear rates the mean dynamic viscosity is higher for higher protein concentrations.

In Figure 3d, the averaged measured viscosity of deionized water at 30 °C depending on the shear rate is shown. For $\dot{\gamma }$ ≥ 3.73 s^−1^, the deviation of measured viscosity from the theoretical reference value of water is less than 4%.

Table 2 summarizes the mean values and 95% confidence intervals of all considered measurements and variations at selected shear rates, on which the following statistical evaluation is based.

The Reynold numbers calculated in the annular gaps at a shear rate of $\dot{\gamma }=1000\;s^{-1}$ are in the range *Re* = 195–271. The critical Reynolds number at which turbulent flows are expected in the annular gab of double gap of double gap measuring systems is $Re_{crit}\geq 1000$.

The mean dynamic viscosity curves presented in Figure 3 were fitted by a Cross model. The predicted Cross parameters for the respective measurements are listed in Table 3. While the Cross time constant was determined to be of the same size for both protein concentrations, a higher zero-shear viscosity $\eta_{0}$ and Cross rate m were determined for a protein concentration of 20 g/L. However, these approximation models are only valid for the analyzed range of shear rates.

### 3.3. Statistic

The measurements at the three different temperatures (35, 37, 39 °C) and a concentration of 20 g/L were approximately normally distributed, as assessed by the Shapiro–Wilk test (*p* > 0.05). At a concentration of 30 g/L and temperatures of 37 and 39 °C, a normal distribution was also observed (*p* > 0.05). However, at a temperature of 35 °C and a concentration of 30 g/L, a deviation from the normal distribution was observed in the lower shear rate range ($\dot{\gamma }$ ≤ 5.18 s^−1^), as assessed by the Shapiro–Wilk test (*p* < 0.05). Above these shear rates, the normal distribution of the values was again established (*p* > 0.05).

A repeated measures ANOVA with a Greenhouse–Geisser correction showed that there is a statistically significant difference between the different temperatures at both protein concentrations (20 g/L: F(1.066, 28.794) = 133.670, *p* < 0.001); 30 g/L: F(1.142, 30.826) = 120.456, *p* < 0.001). Additionally, a repeated measures ANOVA with a Greenhouse–Geisser correction determined that the different protein concentrations at 37 °C showed a statistically significant difference between measurements (F(1.157, 23.132) = 65.734, *p* < 0.001).

A Bonferroni post hoc analysis revealed a significant difference in the comparison between 35 and 37 °C (*p* = 0.002) and between 35 and 39 °C (*p* = 0.02) at a protein concentration of 30 g/L. No statistically significant difference was found when comparing 37 and 39 °C (*p* = 1.000). At a protein concentration of 20 g/L, the measured values did not differ significantly from each other at any temperature. When comparing the different protein concentrations at a temperature of 37 °C, there was a significant difference between the measured values in all combinations. The *p*-values of all comparisons can be found in Table 4. 

**Figure 4:**
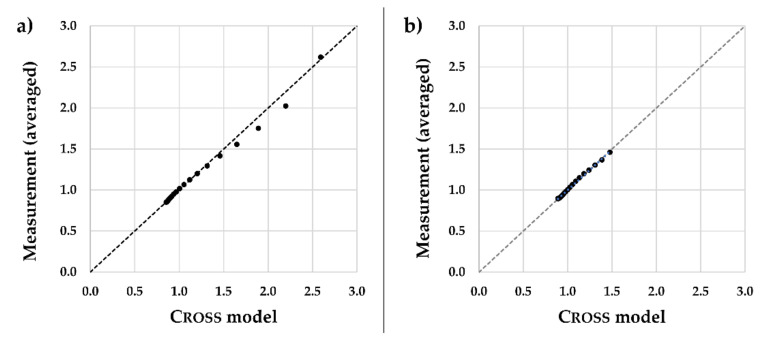
Cross model versus measured dyn. viscosity at a temperature of 37 °C. (**a**) Protein concentration of 20 g/L; (**b**) protein concentration of 30 g/L.

In Figure 4, for protein concentrations of 20 and 30 g/L at a temperature of 37 °C, the results of the Cross model are plotted versus the measured average values. With intra-class-correlation, an ICC = 0.999 with 95% CI = [0.998; 1.000] was obtained at 20 g/L and an ICC = 0.997 with 95% CI = [0.991; 0.999] at 30 g/L. According to Koo et al. [34], an excellent reliability between the measured mean values and the theoretical Cross model can be assumed for both concentrations.

## 4. Discussion

In the field of artificial joint replacement, bovine serum is a common fluid test medium in standardized wear simulations. Nevertheless, little is known about the rheological behavior of this test medium. In particular, for the supporting computer simulations the rheological properties of the fluid such as the dynamic viscosity need to be known for reliable results. Therefore, we determined the rheological behavior of bovine calf serum depending on shear rate, protein concentration and temperature.

First, the density of protein concentrations used in standard joint wear simulations (20 and 30 g/L) was determined since for numerical simulation models the density is needed as an input parameter. Generally, BCS showed a slightly higher density than deionized water. This seems reasonable considering deionized water is the main constituent of the chemical composition. Altogether, an increasing density was found with decreasing temperature and increasing protein concentration, respectively. For a protein concentration of 20 g/L at a temperature of 37 °C a density of 1.0044 g/cm^3^ was found. This is in very good agreement with the observations of Rothammer et al., who found a density of 1.0037 g/cm^3^ at this setting point [33].

The results of the rheometric analysis revealed shear thinning behavior of BCS in the range of low shear rates ($\dot{\gamma }<100\;s^{-1}$), and nearly Newtonian behavior in the range of high shear rates ($\dot{\gamma }\geq 100\;s^{-1}$). Akin to the density, for shear rates $\dot{\gamma }\geq 100\;s^{-1}$, a clear trend of decreasing viscosity with increasing temperature can be seen. However, this does not apply to small shear rates. At 20 and 30 g/L, this correlation only occurs with a shear rate of $\dot{\gamma }\geq 26.8\;s^{-1}$. If the influence of protein concentration is considered in more detail, in the low shear rate range the higher concentration has a lower mean viscosity. For a shear rate of $\dot{\gamma }\geq 26.8\;s^{-1}$, this correlation changed, and higher protein concentrations resulted in a higher dynamic mean viscosity. The phenomenon that the fluids behave differently at lower shear rates than in the higher ones could be due to the sedimentation of proteins in the fluid. Sedimentation is the tendency for particles to settle out from liquids under the influence of weight force. The sedimentation rate depends on the density and size of proteins and the density of the fluid. It can be surmised that due to the increasing density of the fluid with increasing protein concentration, a low sedimentation rate can be expected for high concentrations. If there is no homogeneous distribution of proteins in the fluid, the properties of the fluid could vary. To minimize this influence, a pre-rotation of the fluid was carried out. The exact sedimentation rates of the different proteins and the time and shear rate taken for the mixture to reach homogeneity should be considered in more detail in further studies.

For a protein concentration of 20 g/L and a temperature of 37 °C Rothammer et al. found the viscosity to vary between 0.96 and 1.40 mPa∙s within a shear rate range of 10 to 90 s^−1^ [33]. This is slightly higher than our observations. Contrary to us, Bortel et al. found a constant viscosity of 0.94 ± 0.03 mPa∙s for new born calf serum of 30 g/L protein concentration within a range of $\dot{\gamma }=1\text{–}1000\;s^{-1}$ [35]. However, this value is close to our findings for the range of high shear stresses, though no measuring temperature was stated. Mazzucco et al. also observed Newtonian behavior of bovine serum with 73 mg/mL protein concentration diluted to 40% by volume in distilled water throughout the test range [27]. The viscosity found by Mazzucco et al. (η=1.5 Pa·s) is comparable to our findings at low shear rates. However, no measuring temperature was stated. Nonetheless, our results are in good agreement with the observations found in the literature.

The measurement curves were approximated by Cross models to describe the dependence of the viscosity on the shear rate by a theoretical model. Except for the infinite shear viscosity, the Cross parameters were fitted following Rothammer et al. [33]. For a protein concentration of 20 g/L within a temperature range of 34–40 °C, Rothammer et al. found the zero-shear viscosity varying for 8 mPa∙s to 40 mPa∙s and the Cross time constant varying from 9.5 s to 12.5 s, which is comparable with our ascertainments. However, a Cross rate constant of 0.7–0.75 determined by Rothammer et al. could only be found for a protein concentration of 30 g/L. For a protein concentration of 20 g/L, this parameter was predicted being higher. The infinite shear viscosity was set to the value measured for high shear rates since a Newtonian plateau could be seen at high shear rates.

The Cross model was chosen following rheological models suggested in literature, especially for human synovial fluid. The Cross model is a four-constant-model assuming an inverted sigmoidal shape of the data with a clear distinction between a low-rate plateau and a high-rate plateau. In the present measurements, no low-rate plateau could be achieved, making it difficult to determine the zero-shear viscosity and the Cross time constant C accurately. Here, the Cross parameters were determined empirically using the literature as a reference. Consequently, the parameters do not represent real existing values. Thus, the theoretical models are limited to the considered test range of $\dot{\gamma }=1\text{–}1000\;s^{-1}$. Outside this range, the predicted models may diverge from the real behavior of the fluid. However, the limitation of the considered test range applies to all theoretical models. Beyond that, the direct comparison to Rothammer et al. is critical as the shear rate ranges do not line up exactly.

Measurements of an established hip implant system with a normal head diameter of 36 mm showed radial clearances between 28.8 ± 3.1 µm and 51.1 ± 2.0 µm depending on the material combination [36]. Since mixed lubrication is assumed to be predominant, the adjusted fluid film thickness might be even lower in the range of surface roughness. Furthermore, for ISO 14242 simulations the relative velocity between the main contact point on the head and the insert varied between 10 and 40 mm/s [29]. Since the shear rate was defined as the quotient of relative velocity and gap thickness, shear rates of $\dot{\gamma }\geq 195{\;s}^{-1}$ can be expected from the values determined by Sonntag et al. [36]. Thus, for respective computer simulations, the assumption of Newtonian behavior of BCS might be reasonable. However, the listed determination of the shear rate ranges refers only to the area of hip implant systems. The radial clearances, gap widths and relative velocities that occur in the field of knee implant systems should be considered in more detail in further studies.

Some attention should also given to the quality of measurement data acquisition. First of all, for a constant protein concentration and temperature, different shear rates have been determined during one test run (steady state test) instead of measuring each setting point separately. This was carried out due to time considerations and to define the viscosity as a function of shear rate serving as input data for computer simulations. This lack of independency may, of course, lead to deviations from the true values due to history effects.

To additionally validate the test method, a reference measurement of deionized water at 30 °C was performed. Slight measurement deviations can be seen in the low shear rate range. For a shear rate of $\dot{\gamma }\geq 3.73\;s^{-1}$, an almost optimal agreement was achieved, and the Newtonian behavior of water could be proven. This allowed the validity of the measurement method to be assumed.

However, it is difficult to measure the viscosity of water accurately by commercial systems, especially for low shear rates. Hence, some running-in difficulties could be seen for water, resulting in an average increase in the viscosity at low shear rates, which could be seen for BCS too. However, clear distinctions between the curve shapes of water and BCS can be noticed. Water soon converges to Newtonian behavior after a few measuring points at $\dot{\gamma }=3.73\;s^{-1}$, while BCS shows shear thinning behavior up until $\dot{\gamma }=100{\;s}^{-1}$. Furthermore, for water, some measuring curves also showed a decrease in viscosity towards low shear rates that could not be seen for BCS. Therefore, we assumed a running-in error for water but not for BCS.

Looking at the technical data of the rheometer, the measured torques are at least 28.4 times higher than the lower limit of the rheometer, which is 1 nNm. For BCS, this distance is even greater. So, measurement errors due to measurements close to the system limits are not expected. Furthermore, the deflection angle is higher than 360° for each setting point and only the last 50% of the measurement time was used to generate the respective measured values. This combination should ensure a stable measurement and avoid running-in errors when changing shear rates.

A highest Reynolds number of Re=271 was found for of protein concentration of 20 g/L at 39 °C and $\dot{\gamma }=1000\;s^{-1}$. This is well below the critical Reynolds number $Re_{crit}\geq 1000$. For this reason, turbulent flows should not occur during the measurement. Altogether, our results predict a distinctly lower viscosity for BCS than published for synovial fluid. Healthy synovial fluid obtained post-mortem shows a pronounced shear thinning behavior, varying in average from a zero-shear viscosity $\eta_{0}=104\;\mathrm{mPa}\cdot s$ to a viscosity of $\eta_{1000}=20\;\mathrm{mPa}\cdot s$ at a shear rate of $\dot{\gamma \;}$ = 1000 s^−1^ [24,37,38]. Thus, the viscosity of BCS seems to be of one to two decimal powers lower than the viscosity of synovial fluid. In contrast, Caygill et al. [39] found for healthy synovial fluid viscosities of *η* = 2000–6000 mPa·s at low shear rates and determined viscosities of *η* = 1–7 mPa·s at high shear rates, which is closer to the results we obtained for BCS. However, studies on healthy synovial fluid are difficult. In a healthy joint, only a small amount of fluid is available (about 0.5 mL) and, during post-mortem sampling, most of the fluid is absorbed by the cartilage. Therefore, the obtained amount is not usually sufficient for precise measurements [40]. Reference measurements with bovine synovial fluid show a similar characteristic to human synovial fluid, varying from $\eta_{0}=400\text{–}1000\;\mathrm{mPa}\cdot s$ to $\eta_{1000}=6\text{–}8\;\mathrm{mPa}\cdot s$.

Nonetheless, the composition of synovial fluid varies between individuals. However, in addition to the in vitro substitute bovine serum, further parameters that are decisive for the comparison between in vivo and in vitro conditions have also been examined. In addition to the optical comparison of polyethylene insert damage [41,42,43] and the analysis of PE wear rates [44,45], there is a high level of matching between in vivo and in vitro results when comparing the characteristics of the wear particles [19,46,47,48]. So far, clinical wear mechanisms can only be correctly reproduced in wear studies with bovine calf serum. Therefore, it is prescribed as the standard test medium in ISO standards. These results could not be demonstrated in tests with other test fluids such as phosphate-buffered saline (PBS) or water. The viscosity of bovine calf serum is close to that of water. Attempts to further improve the behavior of bovine calf serum as a lubricant—to make it more viscous by adding hyaluronic acid, which is responsible for the higher viscosity in synovial fluid—have been fruitless. The above could be explained by the presence of different proteins in bovine calf serum, but this has hardly been investigated so far. The behavior of the contained proteins, especially their decomposition or disintegration, could have a high influence on the wear behavior. This influence has to be investigated in more detail at the molecular level in further studies.

The investigations of this study were confined to a detailed analysis of the rheological behavior of bovine calf serum used as fluid test medium in standardized wear simulations of artificial joints. However, comparison of computer simulations and wear testing could provide more information on the accuracy of the obtained results and the validity of considering Newtonian behavior in the range of high shear rates and they might also reveal discrepancies made when using bovine calf serum in place of synovial fluid.

## 5. Conclusions

The determination of the rheological behavior of bovine calf serum, depending on shear rate, protein concentration and temperature, is important for supporting computer simulations and in vitro testing in endoprosthetic joint replacement.

The results showed shear thinning behavior in the range of low shear rates and nearly Newtonian behavior in the range of high shear rates ($\dot{\gamma \;}$= 100–1000 s^−1^). In addition, a bigger effect of protein concentration on the dynamic viscosity can be noticed for small shear rates than for high shear rates. Furthermore, an increasing viscosity with increasing protein concentration and decreasing temperature can be seen for shear rates of $\dot{\gamma \;}\geq 100{\;s}^{-1}$.

Considering the density of bovine calf serum, a higher density was found for the higher protein concentration, while with increasing temperature a slightly decrease in the density could be seen.

In summary, based on the results of the rheological tests carried out, it can be said that the bovine calf serum examined in this study qualifies as a good substitute medium for biomechanical examinations of endoprostheses. Furthermore, on the assumption that for the tribology of endoprosthetic joint replacement shear rates of $\dot{\gamma \;}\geq 100{\;s}^{-1}$ are to be expected, supporting computer simulations may be based on a Newtonian material model.

## Figures and Tables

**Table 1:** Density of 20, 30 and 70 g/L protein concentrations.

Concentration	Temperature (°C)		
(g/L)	35	37	39
20	1.0051 gcm−3	1.0044 gcm−3	1.0037 gcm−3
30	1.0087 gcm−3	1.0072 gcm−3	1.0071 gcm−3
70	1.0208 gcm−3	1.0190 gcm−3	1.0193 gcm−3

**Table 2:** Overview of dynamic viscosity at specific shear rates represented by mean value and 95% confidence intervals.

Shear Rate (s^−1^)	Protein Concentrations and Temperature					
	20 g/L					
	35 °C		37 °C		39 °C	
	MV	95% CI	MV	95% CI	MV	95% CI
1	2.677	[2.309; 3.045]	2.618	[2.198; 3.038]	2.095	[1.347; 2.844]
10	1.099	[1.050; 1.148]	1.122	[1.068; 1.176]	1.043	[0.927; 1.159]
100	0.905	[0.897; 0.9133]	0.887	[0.877; 0.898]	0.846	[0.826; 0.866]
1000	0.883	[0.878; 0.887]	0.850	[0.845; 0.854]	0.819	[0.815; 0.824]
	**30 g/L**					
	**MW**	**95% CI**	**MW**	**95% CI**	**MW**	**95% CI**
1	2.239	[1.765; 2.713]	1.459	[1.269; 1.651]	1.716	[1.441; 1.991]
10	1.216	[1.161; 1.316]	1.065	[1.012; 1.183	1.093	[1.036; 1.151]
100	0.982	[0.967; 0.997]	0.918	[0.905; 0.932]	0.901	[0.888; 0.914
1000	0.940	[0.936; 0.946]	0.896	[0.888; 0.904]	0.869	[0.862; 0.877]

**Table 3:** Predicted parameters for a Cross fit at different temperatures and protein concentrations.

Cross-Parameters	Protein Concentrations and Temperature					
	20 g/L			30 g/L		
	35 °C	37 °C	39 °C	35 °C	37 °C	39 °C
$\mathrm{Zero}-\mathrm{shear}\;\mathrm{viscosity}\;\eta_{0}$ [mPa∙s]	22.0	18.0	11.0	10.0	4.0	6.8
$\mathrm{Infinite}\;\mathrm{shear}\;\mathrm{viscosity}\;\eta_{\infty }$ [mPa∙s]	0.89	0.85	0.82	0.93	0.88	0.86
Cross time constant C [s]	13	13	10	15	11	10
Cross rate constant m	0.95	0.85	0.84	0.7	0.6	0.7

**Table 4:** Results of Bonferroni-adjusted post hoc analysis. * = statistically significant.

Comparison Groups		*p*-Value	MD, 95% CI
Protein concentration—20 g/L			
35 °C	37 °C	1.000	−0.005, 95% CI [−0.16, 0.15]
35 °C	39 °C	0.341	0.097, 95% CI [−0.05, 0.24]
37 °C	39 °C	0.294	0.102, 95% CI [−0.05, 0.25]
**Protein concentration—30 g/L**			
35 °C	37 °C	0.002 *	0.173, 95% CI [0.06, 0.28]
35 °C	39 °C	0.020 *	0.130, 95% CI [0.02, 0.24]
37 °C	39 °C	1.000	−0.043, 95% CI [−0.16, 0.07]
**Fluid temperature—37 °C**			
0 g/L	20 g/L	<0.001 *	−0.361, 95% CI [−0.49, −0.22]
0 g/L	30 g/L	0.001 *	−0.236, 95% CI [−0.37, −0,10]
20 g/L	30 g/L	0.006 *	0.125, 95% CI [0.03, 0.21]

## Data Availability

Data sharing not applicable.
